# Serum concentrations of vitamin D and organ dysfunction in patients with severe sepsis and septic shock

**DOI:** 10.5935/0103-507X.20150063

**Published:** 2015

**Authors:** Fernanda Sampaio Alves, Flavio Geraldo Resende Freitas, Antonio Tonete Bafi, Luciano Cesar Pontes Azevedo, Flavia Ribeiro Machado

**Affiliations:** 1 Discipline of Anesthesiology, Pain, and Intensive Care, Universidade Federal de São Paulo - São Paulo (SP), Brazil.

**Keywords:** Vitamin D, Vitamin D deficiency, Sepsis, Shock, septic, Intensive care

## Abstract

**Objectives:**

To evaluate the serum concentrations of vitamin D and their variations in patients with severe sepsis or septic shock and in control subjects upon admission and after 7 days of hospitalization in the intensive care unit and to correlate these concentrations with the severity of organ dysfunction.

**Methods:**

This case-control, prospective, observational study involved patients aged > 18 years with severe sepsis or septic shock paired with a control group. Serum vitamin D concentrations were measured at inclusion (D0) and on the seventh day after inclusion (D7). Severe deficiency was defined as vitamin D levels < 10ng/ml, deficiency as levels between 10 and 20ng/ml, insufficiency as levels between 20 and 30ng/ml, and sufficiency as levels ≥ 30ng/mL. We considered a change to a higher ranking, together with a 50% increase in the absolute concentration, to represent an improvement.

**Results:**

We included 51 patients (26 with septic shock and 25 controls). The prevalence of vitamin D concentration ≤ 30ng/ml was 98%. There was no correlation between the serum concentration of vitamin D at D0 and the SOFA score at D0 or D7 either in the general population or in the group with septic shock. Patients with improvement in vitamin D deficiency had an improved SOFA score at D7 (p = 0.013).

**Conclusion:**

In the population studied, patients with septic shock showed improvement in the serum concentrations of vitamin D on the seventh day compared with the controls. We also found a correlation between higher vitamin D concentrations and a greater decrease in the severity of organ dysfunction.

## Abstract (pt)

**Objetivo:**

Avaliar as concentrações séricas e a variação de vitamina D em pacientes com sepse grave ou choque séptico e indivíduos controles na admissão e após 7 dias de internação na unidade de terapia intensiva, correlacionando-os com a gravidade da disfunção orgânica.

**Métodos:**

Estudo caso-controle, prospectivo e observacional em pacientes com mais de 18 anos com sepse grave ou choque séptico pareados com grupo controle. Foi realizada dosagem sérica de vitamina D na inclusão (D0) e no sétimo dia (D7). Definiu-se deficiência grave se vitamina D < 10ng/mL, deficiência se entre 10 e 20ng/mL, insuficiência se entre 20 e 30ng/mL e suficiência se ≥ 30ng/mL. Consideramos melhora a modificação para qualquer classificação mais elevada, associada ao incremento de 50% dos valores absolutos.

**Resultados:**

Incluímos 51 pacientes (26 sépticos e 25 controles). A prevalência de concentrações de vitamina D ≤ 30ng/mL foi de 98%. Não houve correlação entre a concentração sérica de vitamina D no D0 e o escore SOFA no D0 ou com sua variação após 7 dias, tanto na população geral quanto nos sépticos. Pacientes com melhora da deficiência tiveram melhora no escore SOFA no D7 (p = 0,013).

**Conclusão:**

Na população estudada, os pacientes sépticos apresentaram melhora das concentrações séricas de vitamina D no sétimo dia em comparação com controles. Encontramos associação entre a melhora das concentrações de vitamina D e a maior redução da intensidade de disfunção orgânica.

## INTRODUCTION

Vitamin D primarily promotes the regulation of the calcium and phosphorus metabolism and is an important regulator of osteomineral parameters. However, the almost universal distribution of vitamin D receptors in human cells suggests that it is involved in systemic homeostasis. Therefore, vitamin D deficiency has been the subject of marked interest by the scientific community and the search for information on its role in critically ill patients is expanding.

Vitamin D regulates both the innate and adaptive immune responses.^(1,2)^ Vitamin D deficiency promotes the deregulation of the immune system, and low serum concentrations of cathelicidins and vitamin D have been found in some critically ill patients with septic shock compared with patients without septic shock. Vitamin D may play a role in the induction of a defense against bacterial and viral agents by stimulating the production of antimicrobial peptides.^(3)^ In addition to its immunomodulatory role, vitamin D appears to suppress inflammatory cytokines, particularly interleukin-6 (IL-6), which trigger systemic inflammatory response syndrome.^(4)^

The prevalence of vitamin D deficiency in critically ill patients with or without sepsis varies between 38% and 100% and is higher than that found in patients hospitalized in non-critical units.^(3,5-7)^ However, the association between a low concentration of vitamin D and worse outcomes is uncertain. Hu et al. have shown that low serum concentrations of vitamin D upon admission to the intensive care unit (ICU) were associated with an increased severity of organ dysfunction.^(8)^ A study involving critically ill patients revealed that the likelihood of death was 1.81-fold higher in individuals with vitamin D concentrations < 20ng/mL.^(7)^ Ginde et al. found a correlation between vitamin D concentrations and the severity of sepsis. Patients with severe sepsis and septic shock had lower vitamin D levels compared with patients with sepsis without organ dysfunction.^(9)^

We conducted a study on critically ill patients to evaluate the serum concentrations of vitamin D and its variations in the first seven days of admission by comparing patients with sepsis and controls. We also tested the correlation of the baseline concentrations of vitamin D and its variations after 7 days of admission with the severity of organ dysfunction.

## METHODS

This case control, prospective, observational study was performed in an ICU with 35 beds with medical and surgical patients. Patients aged > 18 years with severe sepsis or septic shock for less than 48 hours (sepsis group), as defined by the consensus of the American College of Chest Physicians and the Society of Critical Care Medicine of 1992.^(10)^ In addition, we included patients from a control group, at the ratio of 1:1, without clinical signs of infection and with a length of stay in the ICU of < 48 hours (non-sepsis group). The selection of control patients followed the following pairing criteria: gender, maximum age variation of 10 years, and a maximum variation of 3 points in the Sequential Organ Failure Assessment (SOFA) score at admission. In cases of absence of eligible patients in the ICU, the next patient admitted to this unit and who fulfilled the criteria was included. We considered as exclusion criteria in both the groups any of the following characteristics: pregnancy, prior vitamin D supplementation, chronic kidney disease, diseases of bone metabolism, and diseases of calcium metabolism. The study was approved by the Research Ethics Committee of the Universidade Federal de São Paulo under protocol no. 2125/11, and all patients or their legal guardians signed an informed consent form.

All patients from the sepsis and non-sepsis groups were analyzed with respect to demographic variables, comorbidities, diagnosis at hospital admission, diagnosis at ICU admission, Acute Physiology and Chronic Health Evaluation II (APACHE II) score from the time of ICU admission, and SOFA score on the day of inclusion. In the patients with sepsis, the parameters defined included the focus of infection, infectious agents isolated, isolation sites, infection characteristics (community-acquired, hospital-acquired, and ICU-acquired), and infection category (severe sepsis and septic shock).

The patients were included within the first 48 hours of the development of organ dysfunction. At this time (D0), blood samples were collected for the administration of vitamin D in the form of 25-hydroxyvitamin D (25(OH)D), ionized calcium, magnesium, urea, and creatinine. Another serum sample was collected on the seventh day after inclusion (D7) from the patients who remained in the ICU for analysis of the same laboratory parameters. To determine the serum concentrations of vitamin D, 1mL of whole blood was collected in EDTA tubes. The samples were promptly chilled to -4°C and stored at -80°C when they were not immediately analyzed. The analysis was performed using the immunoassay method involving automated electrochemiluminescence developed by Roche Diagnostics GmbH (Mannheim, Germany) on a Cobas 6000 modular analyzer. The reported coefficient of variation of the method is 1.9% - 5.5%. Severe deficiency was defined as vitamin D concentrations below 10ng/mL; deficiency was defined as concentrations ​​between 10 and 20ng/ml; insufficiency was defined as concentrations between 20 and 30ng/mL; sufficiency was defined as concentrations ≥30ng/mL.^(11,12)^ To calculate the prevalence of vitamin D deficiency, any value lower than the reference value (30ng/mL) was used. The evaluation of the improvement of vitamin D concentrations between D0 and D7 was defined *a priori*. Changes from lower concentrations to higher concentrations, together with an increase of 50% in the absolute concentrations of vitamin D, represented improvement. All other situations were considered to represent worsening. We also calculated the difference between the vitamin D concentrations at D0 and D7 (∆vitD) in the sepsis and control groups. The patients who were not evaluated at D7 were excluded from this analysis.

In addition to the determination of the SOFA score on the day of inclusion, clinical and laboratory data were collected to determine the SOFA score on the seventh day of inclusion (SOFA D7). We calculated the difference between the SOFA scores at D0 and D7 (∆SOFA) in the sepsis and non-sepsis groups. An improvement in the SOFA score was represented by a decrease in the score by one or more points between D0 and D7. Worsening was represented by no changes in the SOFA score between the evaluation times or when there was an increase of ≥ 1 point in ∆SOFA.

The mortality rates and period of hospitalization in the ICU and hospital were determined for all patients.

### Statistical analysis

The sample size was calculated to determine the correlation between the vitamin D concentrations and the SOFA score. The null hypothesis was a correlation with r = 0.4 and the alternative hypothesis was a correlation with r = 0.7. Considering a power of 80% and an alpha error of 0.05, the sample size required for the study was 44 patients. Considering the potential non-normal distribution of the variable, this number was adjusted to 50 patients.

The results of continuous variables were expressed as the mean ± standard deviation or median (25^th^ to 75^th^ percentile) according to their distribution. The normality of the distribution was assessed using the Kolmogorov-Smirnov test and the homogeneity of variance was assessed using Bartlett's test.

The variables evaluated were compared between the groups using Pearson's chi-square test and the Mann-Whitney test, given the non-normal distribution of the variables. We compared the concentrations of vitamin D at D0 and D7 and the SOFA scores in the sepsis and non-sepsis groups using the paired Wilcoxon test.

Spearman's correlation coefficient was used to evaluate the degree of the correlation between the quantitative variables. In the patients with and without sepsis, we correlated the vitamin D concentrations at D0 with the variations in its concentrations, the severity scores (SOFA, ∆SOFA, and APACHE II), laboratory parameters at D0 (calcium, magnesium, creatinine, and urea), and mortality.

All statistical calculations were performed using the Statistical Package for the Social Sciences (SPSS) version 22 for Windows. A p-value < 0.05 was considered significant.

## RESULTS

Between February 2012 and October 2013, 51 patients were evaluated, including 26 patients with sepsis and 25 patients without sepsis. Data at D7 were not available in 9 patients because 4 patients were discharged and 5 died before D7. Table 1 shows the clinical characteristics of the patients according to the groups. The study population predominantly comprised men and patients who came from the surgical center. Immunosuppression was more common among the patients with sepsis than among the controls (p = 0.041). Most patients with infections met the criteria for septic shock (69%). The main focus of infection was pulmonary (46%), and most patients (54%) acquired the infection during hospitalization.

**Table 1:** Clinical characteristics of the study population according to the presence or absence of sepsis

Characteristics	With sepsis	Without sepsis	p value*
	(N = 26)	(N = 25)	
Age (years)	53 (38 - 63)	53 [35 - 67]	0.850
Gender, male	16 (62)	14 (56)	0.688
Ethnicity			0.200
Caucasian	12 (46)	11 (44)	
Black	12 (46)	9 (36)	
Asian	2 (08)	0	
Mixed	0	5 (20)	
Comorbidities			
Hypertension	7 (27)	8 (32)	0.691
Diabetes mellitus	3 (12)	3 (12)	0.959
Heart failure	3 (12)	1 (4)	0.317
Liver failure	3 (12)	2 (8)	0.671
Neoplasia	4 (15)	7 (28)	0.274
Immunosuppression	4 (15)	0	0.041
COPD	1 (4)	0	0.322
AMI	1 (4)	1 (4)	0.977
Stroke	1 (4)	1 (4)	0.977
Others	5 (19)	2 (8)	0.244
APACHE II (points)	15 [12 - 18]	15 [12 - 20]	0.460
Diagnosis at admission			0.000
Sepsis	24 (85)	0	
Polytrauma	2 (8)	10 (40)	
Large surgery	0	10 (40)	
Others	2 (7)	5 (20)	
Hospital service of origin			0.010
Ward	8 (31)	0	
Emergency	6 (23)	1 (4)	
Surgical center	12 (46)	23 (92)	
Another service	0	1 (4)	
Type of hospital			0.000
Clinical	13 (50)	2 (8)	
Surgical	13 (50)	23 (92)	
Hospital mortality	7 (27)	5 (20)	0.560
Length of stay in ICU (days)	13.5 (7.7 - 22.5)	12 (9.0 - 20.5)	0.821

COPD - chronic obstructive pulmonary disease; AMI - acute myocardial infarction; APACHE - Acute Physiological and Chronic Health Evaluation; ICU - intensive care unit. The results are expressed as numbers (%) or medians [25% to 75% percentile].

* Chi-square and Mann-Whitney U tests.

The prevalence of low vitamin D concentrations was 98%. At admission, we found that severe vitamin D deficiency (< 10ng/mL) occurred in 69.2% of patients with sepsis but in only 48% of patients without sepsis. Sufficient levels were found only in the control group. However, this difference in distribution between the groups was not significant. Similarly, no significant difference was found between the patients with sepsis and controls when the vitamin D concentrations were analyzed without classification at both D0 and D7. However, the variation in vitamin D concentrations was higher in the group with sepsis compared with the group without sepsis, with an increase in these levels at D7 (Table 2).

**Table 2:** Laboratory parameters and organ dysfunction in the patients according to groups

Characteristics	Sepsis	Without sepsis	p value*
	(N = 26)	(N = 25)	
Vitamin D at D0 (ng/ml)	8.8 [4.5 - 11.3]	11.5 [5.0 - 14.8]	0.261
Vitamin D at D7 (ng/ml)	10 [5.0 - 19.3]†	11.6 [5.5 - 15.4]†	0.771
∆vit D	1.4 [-0.1 - 9.5]	0 [-5.6 - 1.9]	0.017
∆vit D (%)	109.9 [9.1 - 215.4]	100 [60.0 - 142.4]	0.011
Classification of vitamin D at D0			0.371
< 10ng/ml	18 (69.2)	12 (48.0)	
≥ 10ng/mL to < 20ng/ml	6 (23.1)	8 (32.0)	
≥ 20ng/mL to < 30ng/ml	2 (7.7)	4 (16.0)	
≥ 30ng/mL	0	1 (4.0)	
Classification of vitamin D at D7			0.506
< 10ng/ml	11 (47.8)	8 (42.1)	
≥ 10ng/mL to < 20ng/ml	8 (34.8)	10 (52.6)	
≥ 20ng/mL to < 30ng/ml	3 (13.0)	1 (5.3)	
≥ 30ng/mL	1 (4.3)	0 (0)	
SOFA score at D0 (points)	6 [5 - 9]	7 [4 - 9]	~ 1.000
SOFA score at D7 (points)	1 [0 - 4]	7 [3 - 10]	0.004
∆SOFA	-4 [-6 to -1]	-1 [-5 to 2]	0.024
Calcium at D0 (mmol/L)	1.1 [1.1 - 1.2]	1.1 [1.1 - 1.2]	0.610
Calcium at D7 (mmol/L)	1.1 [1.1 - 1.2]	1.1 [1.1 - 1.2]	0.762
Magnesium at D0 (mg/dL)	1.9 [1.6 - 2.2]	1.9 [1.8 - 2.1]	0.932
Magnesium at D7 (mg/dL)	1.8 [1.7 - 2.0]	2 [1.8 - 2.3]	0.002
Urea at D0 (mg/dL)	32.5 [20 - 63]	32 [20 - 40]	0.480
Urea at D7 (mg/dL)	47 [23 - 77]	43 [23 - 63]	0.820
Creatinine at D0 (mg/dL)	0.7 [0.5 - 1.2]	0.8 [0.6 - 1.3]	0.396
Creatinine at D7 (mg/dL)	0.8 [0.4 - 1.3]	0.8 [0.5 - 1.8]	0.536

D0 - day of admission; D7 - 7 days after admission; Avit D - variation between vitamin D concentrations at D7 and DO; SOFA - Sequential Organ Failure Assessment; ASOFA -variation in the SOFA between D7 and D0. Data for the seventh day refer to 49 patients. The results are expressed as numbers (%) or medians (25% to 75% percentile).

* Chi-square and Mann-Whitney U tests;

† Paired Wilcoxon test - vitamin D concentrations: group with sepsis, p = 0.007; group without sepsis, p = 0.478; SOFA score: group with sepsis, p < 0.0001, group without sepsis, p = 0.330.

There was no significant correlation between the vitamin D concentrations at D0 and SOFA score at admission or ∆SOFA for the general population (r = 0.178, p = 0.211, for SOFA at admission; r = 0.009, p = 0.954 for ∆SOFA) or for the septic group (r = 0.107, p = 0.604 for SOFA at admission; r = 0.148, p = 0.500 for ∆SOFA). The analysis of the correlation of the baseline levels of vitamin D with other variables indicated a weak positive correlation with the baseline levels of magnesium. The analysis of a possible correlation between ∆vitD and ∆SOFA indicated no significant correlation. A lack of a correlation was also observed with other variables, including age, APACHE II, calcium levels at D0, urea levels at D0, and creatinine levels at D0 (Table 1S of the electronic supplementary material).

There was improvement in vitamin D deficiency in only 7 patients, whereas in 35 patients, these levels were unchanged or worsened. In the subgroup of patients with an improvement in the classification of vitamin D, we observed a trend towards a higher prevalence of sepsis (p = 0.071). In addition, the patients with improved classification had an improved SOFA score at D7 (Table 3). There was no association with mortality.

**Table 3:** Clinical characteristics of the population, laboratory parameters, and severity of organ dysfunction according to changes in the classification of vitamin D levels.

Characteristics	Improvement of the classification of vitamin D^a^	Worsening of the classification of vitamin D*	p value†
	(N = 7)	(N = 35)	
Age (years)	49 [32 - 62]	51 [36 - 67]	0.426
APACHE II (points)	13 [11 - 18]	14 [12 - 19]	0.716
Group			0.071
With sepsis	6 (85.7)	17 (48.6)	
Without sepsis	1 (14.3)	18 (51.4)	
Gender, male	6 (85.7)	20 (57.1)	0.155
Diagnosis at admission			0.537
Sepsis	5 (71.4)	15 (42.8)	
Polytrauma	1 (14.3)	10 (28.6)	
Large surgery	1 (14.3)	4 (11.4)	
Others	0	6 (17.2)	
Hospital service of origin			0.589
Ward	0 (00)	5 (14.3)	
Emergency	2 (28.5)	5 (14.3)	
Surgical center	5 (711.5)	24 (68.6)	
Other services	0	1 (2.8)	
Type of hospital			0.706
Clinical	2 (28.5)	10 (28.6)	
Surgical	5 (71.5)	25 (71.4)	
Vitamin D at D0 (ng/ml)	9.0 [6.7 - 9.5]	9.1 [4.5 - 14.1]	0.668
SOFA score at D0	6 [6 - 8]	7 [4 - 9]	0.921
∆SOFA score at D7	-6 [-6 to -5]	-2 [-5 to 0]	0.013
Calcium at D0 (mmol/L)	1.13 [1.08 - 1.16]	1.14 [1.11 - 1.2]	0.319
Magnesium at D0 (mg/dL)	1.6 [1.6 - 1.9]	1.9 [1.7 - 2.1]	0.092
Creatinine at D0 (mg/dL)	0.72 [0.57 - 0.89]	0.74 [0.55 - 1.32]	0.597
Urea at D0 (mg/dl)	22 [16 - 25]	32 [20 - 59]	0.217
Hospital mortality	0	7 (20)	0.195

APACHE - Acute Physiology and Chronic Health Evaluation; DO - day of admission; SOFA - Sequential Organ Failure Assessment; ASOFA - variation in SOFA between D7 and DO; D7 - seventh day of admission;

* Classification of vitamin D concentrations: < 10ng/m; > 10ng/mL to < 20ng/ml; > 20ng/mL to < 30ng/ml; > 30ng/mL. Improvement in the classification of vitamin D levels: change in the classification of vitamin D to higher values between D0 and D7 together with an increase in the vitamin D concentration > 50% between D7 and D0; Worsening or maintenance of the classification of vitamin D levels: change in the classification of vitamin D to lower values, absence of changes in the classification between D0 and D7, or increase in the vitamin D concentrations < 50% between D7 an D0. The results are expressed as numbers (%) or medians [25% to 75% percentile].

† Chi-square and Mann-Whitney U tests.

## DISCUSSION

Our study showed a high prevalence of low vitamin D levels (98%) in critically ill patients. Although more patients with sepsis had severe vitamin D deficiency, there was no significant difference in the prevalence of low concentrations of vitamin D between the patients with and without sepsis. However, the vitamin D concentrations improved after 7 days in patients with sepsis more frequently than in patients without sepsis. The baseline vitamin D concentrations were not correlated with the severity of organ dysfunction. However, the variation in these levels after 7 days was associated with improved severity of organ dysfunction.

Despite the different cut-off points for the definition of vitamin D deficiency, most studies showed a high prevalence in 100% of the cases in some samples of critically ill patients.^(3,5,6)^ Lee et al. found that the median concentrations of vitamin D varied between 4 and 16 ng/mL,^(6)^ similar to the concentrations found in our study, in which the median level was < 10ng/mL at inclusion. Most studies indicate the presence of low levels of vitamin D within the first hours of critical illness.^(3,7,13)^ However, we cannot confirm whether such levels reflect previous vitamin D deficiency or whether they are a consequence of critical illness.

Similar to the results of our study, Jeng et al. found no significant differences in vitamin D concentrations in patients with sepsis compared with critically ill patients in the ICU.^(3)^ However, other studies report that vitamin D deficiency is more common in patients with sepsis.^(2,14)^ One of the mechanisms proposed for this association is a more frequent decrease in the serum concentrations of the proteins that transport vitamin D in patients with sepsis, as proposed by Watkins et al.^(2)^ Because the matching criteria were predefined according to severity, we did not find this difference in our study sample. In contrast, similar to the results of a cohort study conducted in 2012,^(15)^ we demonstrated that severe vitamin D deficiency (levels < 10ng/ml) was present in 69% of the patients with sepsis but in only 48% of the controls.

In the intensive care setting, a correlation between low concentrations of vitamin D and worse outcomes has been suggested. Hu et al. demonstrated that low concentrations of vitamin D at admission were associated with increased severity of organ dysfunction.^(8)^ Vitamin D deficiency has also been associated with longer hospital stays, an increased tendency toward ICU-acquired infections,^(16)^ and an increased frequency of new episodes of sepsis.^(14,17)^ In our sample, low concentrations of vitamin D at D0 were not correlated with the increased severity of organ dysfunction at D0 and D7 in patients with sepsis or the controls. However, this finding may be a result of the small sample size.

The sequential assessment of vitamin D concentrations during hospitalization has been relatively unexplored. In three studies, this assessment was conducted in 7 to 10 days and indicated the persistence of low levels of vitamin D throughout hospitalization; these results are similar to those found in our study population.^(4,16,18)^ However, the correlation between the positive variation in the vitamin D concentrations and better outcomes has not been established. In our study, we found no association between ∆vitD and ∆SOFA. However, the analysis of our results using a classification system clearly indicates that the patients whose vitamin D concentrations increased exhibited a greater decrease in the level of organ dysfunction. Although this study was not designed to evaluate potential causes, some hypotheses may explain these findings. Vitamin D plays a key role in the modulation of inflammatory cytokines that trigger and perpetuate systemic inflammatory response syndrome, and vitamin D deficiency is associated with the worsening of metabolic and immune dysfunction in critically ill patients.^(1)^ Higher vitamin D concentrations, even without reaching normal values, may be implicated in improved immunomodulatory regulation and slower progression to organ dysfunction. However, considering the study design, we could not assess a cause and effect relationship, i.e., we only found an association between the improvement of the clinical conditions of the patients and the improvement of serum concentrations of vitamin D.

We found a weak positive correlation between the concentrations of magnesium and vitamin D on admission. Several steps of the process of vitamin D metabolism utilize magnesium as a cofactor. According to some studies, the prevalence of hypomagnesaemia in critically ill patients can range between 11% and 61%, and its correlation with worse outcomes is controversial.^(19,20)^ Deng et al. demonstrated that patients with higher concentrations of magnesium have a lower risk of having poor/inadequate concentrations of vitamin D. In addition, patients with higher concentrations of magnesium have a negative correlation between circulating concentrations of vitamin D and mortality.^(21)^

One of the strengths of our study included the use of a control group matched by predetermined severity criteria, which allowed for the formation of a more homogeneous study population compared with previous studies. We also used a scoring system that was validated for the analysis of organ dysfunction, which made the comparison between the groups more reliable. Furthermore, we performed a continuous assessment of vitamin D levels by measuring its concentrations after 7 days of admission, thereby allowing one to test the correlation between variations in these levels and clinical and laboratory outcomes.

However, this study has some limitations. First, although it was based on sample size calculations, the number of patients may not have been sufficient for the analysis of all the objectives of the study. The isolated use of vitamin D concentrations to assess the functional profile of vitamin D may be considered a limiting factor, and there are inherent limitations in the measurement technique used. In addition, a second control group of non-critical patients would help to elucidate the impact of vitamin D deficiency in the critically ill population compared with other hospitalized patients. Because of the study design, the associations found did not support a causal relationship. Furthermore, in nine patients, it was not possible to measure vitamin D levels on the seventh day, which may have restricted the evaluation of the association between variations in the concentration of vitamin D and the severity of organ dysfunction.

## CONCLUSION

The prevalence of vitamin D deficiency was high in critically ill patients with and without sepsis. Patients with sepsis showed a marked improvement in the vitamin D concentrations on the seventh day compared with patients without sepsis. There was no correlation between baseline concentrations of vitamin D, variations in the concentration after 7 days of admission, and the severity of early organ dysfunction. In addition, there was no correlation between variations in the concentration of vitamin D and variations in the severity of organ dysfunction 7 days after admission. However, there was an association between improvement in the classification of vitamin D deficiency and a greater decrease in the severity of organ dysfunction.
