# Supplementary material for: Serum concentrations of vitamin D and organ dysfunction in patients with severe sepsis and septic shock
Source: Rev Bras Ter Intensiva. 2015 Oct-Dec;27(4):376–82. doi: 10.5935/0103-507X.20150063 (PMC4738824; doi:10.5935/0103-507X.20150063)
Supplement: Supplementary file 1 [file rbti-27-04-0376-suppl01.pdf]

Serum concentrations of vitamin D and organ dysfunction in patients with severe sepsis and septic shock

Concentrações séricas de vitamina D e disfunção orgânica em pacientes com sepse grave e choque séptico

Fernanda Sampaio Alves[1], Flavio Geraldo Resende Freitas[1], Antonio Tonete Bafi[1], Luciano Cesar Pontes Azevedo[1], Flavia Ribeiro Machado[1]

Table 1S - Correlation between vitamin D concentrations, variations in the concentrations, severity scores, and laboratory parameters

Variables	General population (N = 51)		Patients with sepsis (N = 26)	
	R	p value	R	p value
Vitamin D at D0				
SOFA	0.178	0.211	0.107	0.604
ΔSOFA	0.009	0.954	0.148	0.500
Age	-0.228	0.108	-0.143	0.486
APACHE II	0.094	0.513	0.093	0.653
Calcium at D0	-0.097	0.497	-0.066	0.750
Magnesium at D0	0.387	0.005	0.494	0.010
Urea at D0	0.047	0.741	-0.113	0.581
Creatinine at D0	0.129	0.367	-0.056	0.785
ΔvitD				
SOFA	0.095	0.549	0.158	0.471
ΔSOFA	-0.290	0.063	-0.306	0.155
Age	-0.122	0.440	-0.307	0.155
APACHE II	0.650	0.682	0.226	0.300
Calcium at D0	-0.196	0.213	-0.108	0.622
Magnesium at D0	0.378	0.140	-0.365	0.086
Urea at D0	-0.266	0.890	-0.225	0.303
Creatinine at D0	-0.530	0.739	0.215	0.325

D0 - day of admission; APACHE - Acute Physiology and Chronic Health Evaluation; SOFA - Sequential Organ Failure Assessment; ΔSOFA - variation in SOFA between D7 and D0; ΔvitD - variations in the vitamin D concentrations between D7 and D0. Spearman's correlation.
